# Supplementary material for: Antileukemic Cell Proliferation of Active Compounds from Kaffir Lime (Citrus hystrix) Leaves
Source: Molecules. 2020 Mar 12;25(6):1300. doi: 10.3390/molecules25061300 (PMC7144100; doi:10.3390/molecules25061300)
Supplement: Supplementary file 1 [file molecules-25-01300-s001.pdf]

SUPPLEMENTARY DATA

Antileukemic Cell Proliferation of Active Compounds from Kaffir Lime (*Citrus hystrix*) Leaves

Songyot Anuchapreeda [1,2,3,4,#,*], Fah Chueahongthong [1,#], Natsima Viriyaadhammaa [1], Pawaret Panyajai [1], Riki Anzawa [2], Singkome Tima [1,3,4], Chadarat Ampasavate [5], Aroonchai Saiai [6], Methee Rungrojsakul [7], Toyonobu Usuki [2,*] and Siriporn Okonogi [4,5,*]

[1] Department of Medical Technology, Faculty of Associated Medical Sciences, Chiang Mai University, Chiang Mai 50200, Thailand; fahmyfah@hotmail.com (F.C.); fai.natsima@gmail.com (N.V.); panyajaip@gmail.com (P.P.); singkome@gmail.com (S.T.)

[2] Department of Materials and Life Sciences, Faculty of Science and Technology, Sophia University, Tokyo 102-8554, Japan; r-anzawa-4g3@eagle.sophia.ac.jp

[3] Cancer Research Unit of Associated Medical Sciences (AMS CRU), Faculty of Associated Medical Sciences, Chiang Mai University, Chiang Mai 50200, Thailand; singkome@gmail.com

[4] Research Center of Pharmaceutical Nanotechnology, Chiang Mai University, Chiang Mai, 50200, Thailand

[5] Department of Pharmaceutical Sciences, Faculty of Pharmacy, Chiang Mai University, Chiang Mai 50200, Thailand; aimchadarat@windowslive.com

[6] Department of Chemistry, Faculty of Science, Chiang Mai University, Chiang Mai 50200, Thailand; saiai_aroonchai@hotmail.com

[7] College of Alternative Medicine, Chandrakasem Rajabhat University, Bangkok 10900, Thailand; mathewhor@hotmail.com

* Correspondence: sanuchapreeda@gmail.com (S.A.); t-usuki@sophia.ac.jp (T.U.); okng2000@gmail.com (S.O.); Tel.: +66-5394-9237 (S.A.); +81-3-3238-3446 (T.U.); +66-5394-1512 (S.O.)

[#] The first two authors (S.A. and F.C.) contributed as co-first author.

Academic Editor: Rob Keyzers

Received: 7 February 2020; Accepted: date; Published: date**Abstract:** Kaffir lime (*Citrus hystrix*) is a plant member of family Rutaceae, and its leaves are commonly used in folk medicine. The present study explores antileukemic effects of the extracts and purified active compounds from the leaves. The antileukemic activity was investigated via inhibition of Wilms' tumor 1 (WT1), which is a protein that involves in leukemic cell proliferation. In addition, the compounds were investigated for their effects on *WT1* gene expression using real time RT-PCR and Western blotting. Cell cycle arrest and total cell number were investigated using flow cytometry and trypan blue exclusion method, respectively. The results demonstrated that the hexane fractionated extract had the greatest inhibitory effect on *WT1* gene expression of many leukemic cell lines and significantly decreased WT1 protein levels of K562 cells (representative of the leukemic cells), in a dose- and time-dependent manner. Subfraction No. 9 (F9) after partial purification of hexane fractioned extract showed the highest suppression on WT1 protein and suppressed cell cycle at G2/M. The organic compounds were isolated from F9 and identified as phytol and lupeol. The bioassays confirmed antiproliferative activities of natural products phytol and lupeol. The results demonstrated anticancer activity of the isolated phytol and lupeol to decrease leukemic cell proliferation.

Keywords: kaffir lime; phytol; lupeol; Wilms' tumor 1; leukemia; antiproliferation

Table S1. Percent yield of partial purification fractions of hexane extract (% w/w) after vacuum column Chromatography

Sub-fraction No.	Solvent Hexane:Dichloromethane	Weight	% Yield
1	100:0	0	0
2	100:0	0.62	7.75
3	97.5:2.5	0.55	6.88
4	95:5	0.28	3.5
5	90:10	0.29	3.63
6	80:20	0.4	5
7	70:30	0.96	12
8	50:50	0.96	12
9	20:80	0.78	9.75
10	10:90	0.71	8.88
11	0:100	0.3	3.75
	Dichloromethane:Methanol		
12	95:5	0.05	0.63
13	90:10	0.96	12.00
		6.86	85.75
	Column chromatography		
Purified sub-fraction No. 9		0.051	6.5

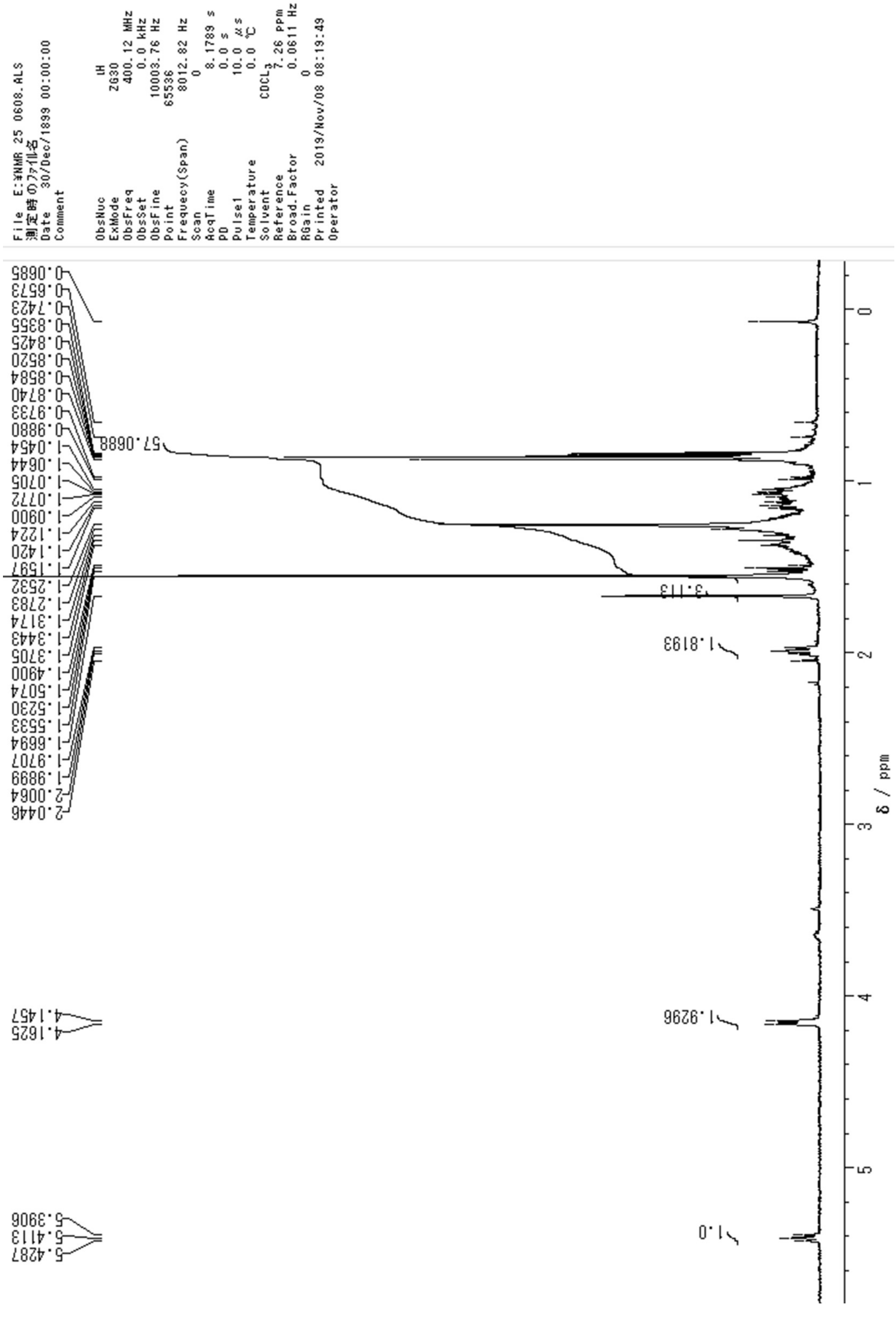

Figure S1. ^{1}H NMR spectrum of isolated phytol in $CDCl_3$.

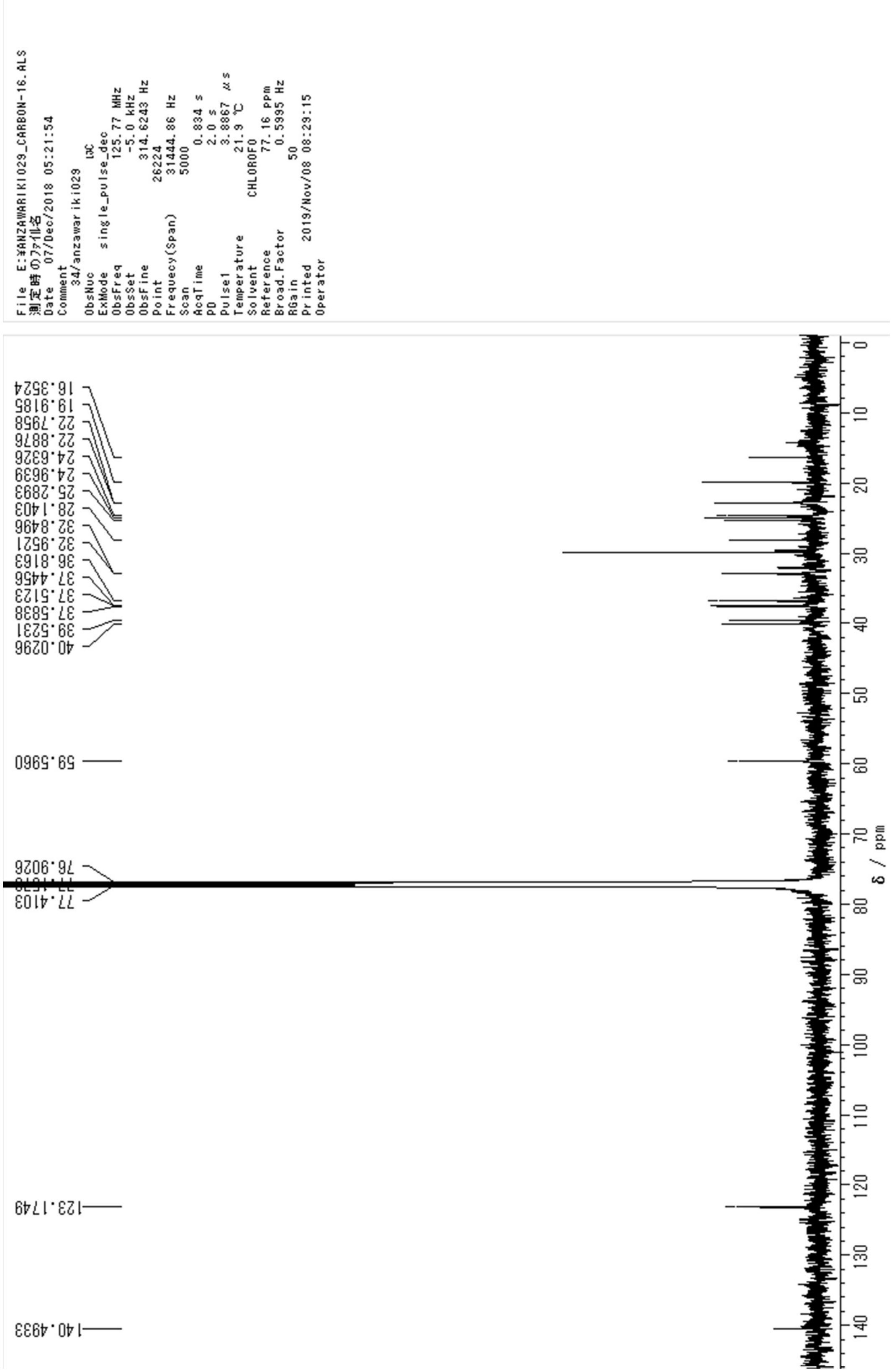

Figure S2. ^{13}C NMR spectrum of isolated phytol in $CDCl_3$.

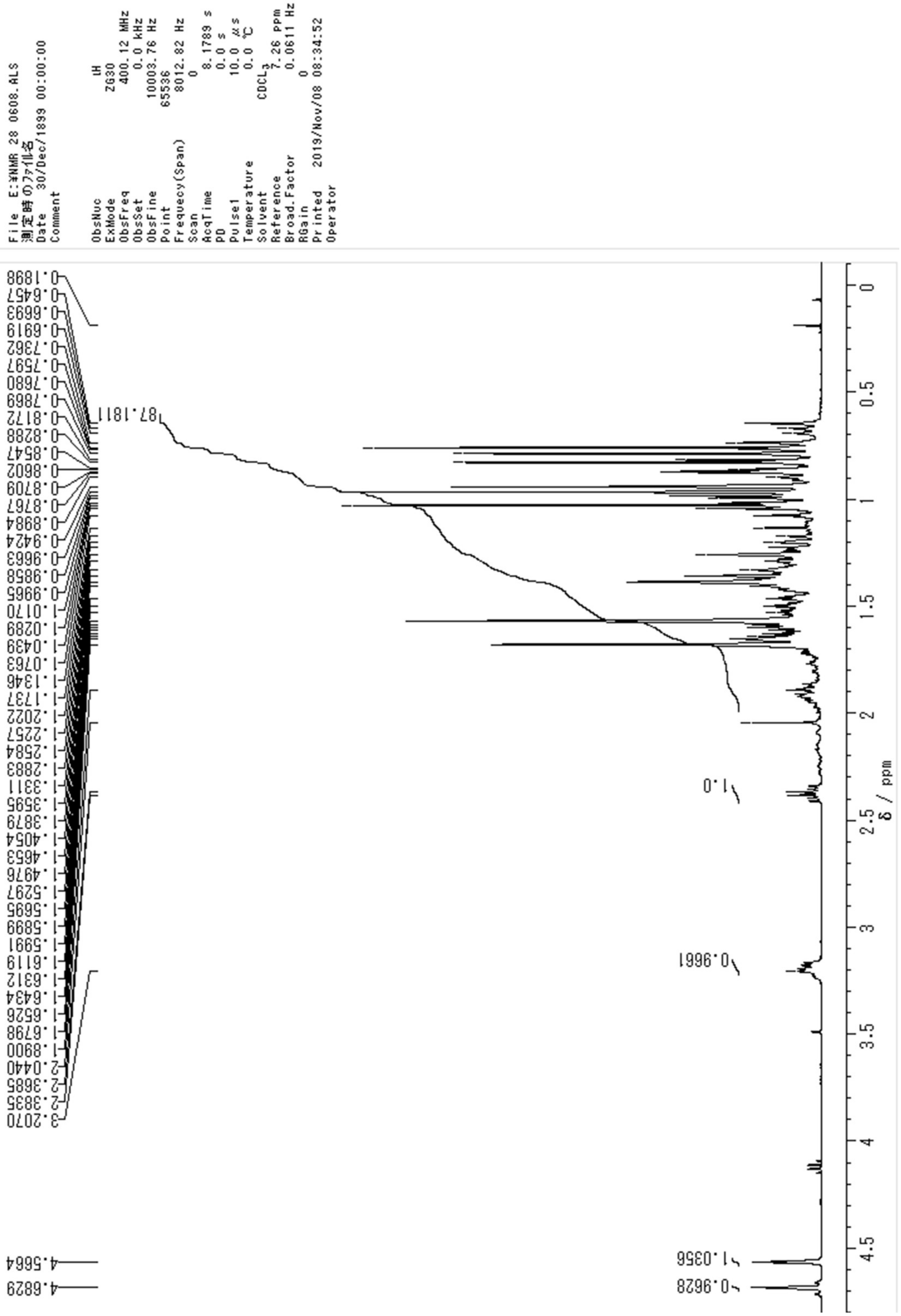

Figure S3. ^{1}H NMR spectrum of isolated lupeol in $CDCl_3$.

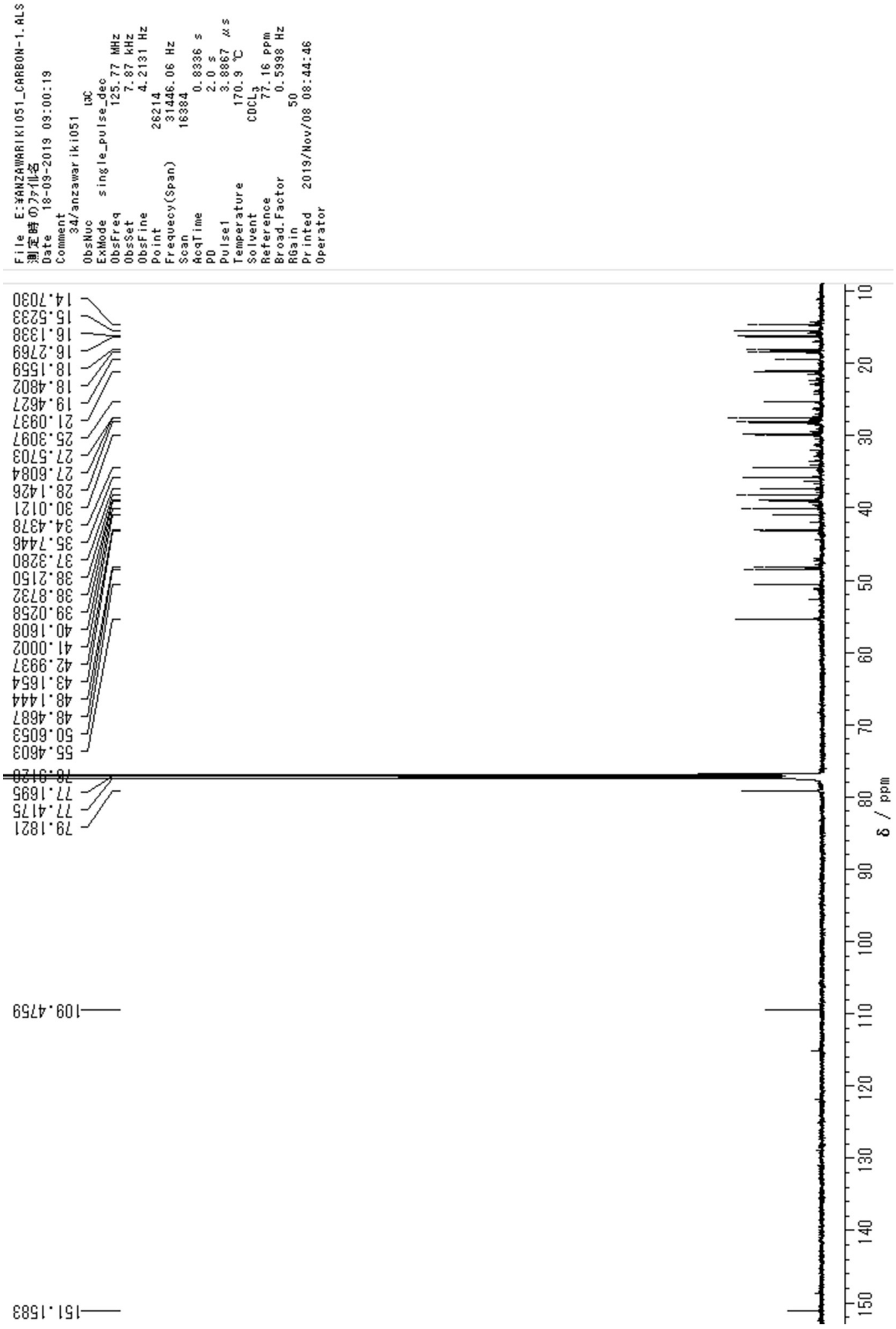

Figure S4. ^{13}C NMR spectrum of isolated lupeol in $CDCl_3$.
